# Effect of EAP Psychological Intervention on Improving the Mental Health of Medical Workers Under the Novel Coronavirus Epidemic in China

**DOI:** 10.3389/fpubh.2021.649157

**Published:** 2021-07-28

**Authors:** Jun Xu, Xia Liu, Yundan Xiao, Xiaohui Fang, Yingsheng Cheng, Jinping Zhang

**Affiliations:** The Sixth People's Hospital Affiliated to Shanghai Jiaotong University, Shanghai, China

**Keywords:** novel coronavirus, employee assistance program, EAP psychological intervention, SCL-90 psychological evaluation, medical workers, psychological changes

## Abstract

**Background:** Due to the novel coronavirus epidemic, medical workers are under immense psychological pressure. As such, the East Campus of Shanghai Sixth People's Hospital actively adopted the Symptoms Checklist 90 (SCL-90) to evaluate the mental health of hospital staff before and after the psychological intervention from the Employee Assistance Program (EAP).

**Methods:** Medical workers from the East Campus of Shanghai Sixth People's Hospital were recruited for this study. Psychological evaluations were conducted using the SCL-90, with a score of >160 regarded as a positive result, or in other words, an indication of abnormal psychological symptoms. The EAP adopted different forms of psychological interventions for healthcare professionals, and participation in these measures was entirely voluntary. Medical workers completed the SCL-90 again after participating in the psychological intervention, and we analyzed the changes between their two assessments.

**Results:** Of the 1,198 total medical staff present at the hospital, 844 participated in the initial survey, while only 652 completed the survey a second time (i.e., post-psychological intervention). Multivariate logistic regression analysis found that the psychological status of hospital staff was correlated with gender, education background, and fertility status (*P* < 0.05). The results showed that, compared with women, men's mental health status was better, with an OR value of 0.598 (0.372–0.962). Groups with high school, junior high school, and below education levels were at higher risk of psychological problems, with OR values of 23.655 (2.815–198.784) and 9.09 (2.601–31.801), respectively. Administrative occupations and having two or more children were protective factors for mental health, and the OR values were 0.400 (0.175–0.912) and 0.327 (0.152–0.703), respectively.

Following the psychological intervention, we found that the mental health of hospital workers improved, as indicated by their second SCL-90 evaluations, although the proportion of medical staff willing to participate in the second evaluation was lower than the initial assessment. There were differences in the SCL-90 scores among different occupations, and there were also differences in the scores of employees of different occupations who had participated in the two evaluations. The employees of different positions who participated in the two evaluations were matched and analyzed and found that the depression and anxiety of the doctor group were significantly reduced. In the nursing group, the total score, somatization, interpersonal sensitivity, depression, and anxiety were significantly reduced. In the medical technician group, depression, anxiety, and paranoia were reduced considerably. Among office staff, no significant differences were found. Among workers, the total score, depression, and anxiety were significantly reduced.

**Conclusion:** Hospitals have the potential to alleviate and reduce the psychological pressure placed on medical staff members through the EAP, which can actively adopt intervention and guidance measures. The findings of this study have important implications, as reducing abnormal psychological symptoms of healthcare professionals can be helpful in the fight against the coronavirus epidemic.

## Introduction

In December 2019, the novel coronavirus outbreak occurred in China, and the virus has now been listed as a first-level medical prevention and control event at the national level (1). The World Health Organization (WHO) named the virus “novel coronavirus,” that is, CoV-2019. The pathogen is primarily transmitted by respiratory droplets and is highly contagious (2). Infection rates are increasing exponentially worldwide, and the mortality rate is high in some countries. However, the virus was well-controlled in China because public health authorities responded promptly, implementing strict control measures throughout the country (3). Previously, China has faced outbreaks of severe acute respiratory syndrome (SARS) and bird flu, which led to psychological disorders in a large number of people (4). Thus, given previous experience in managing severe disease outbreaks such as SARS (3), China has prioritized mental health counseling from the beginning of the novel coronavirus epidemic. Early on in the disease outbreak, when the numbers of confirmed cases and deaths were increasing, many members of the public (5), especially medical staff, experienced psychological symptoms such as anxiety and tension (6). If mental health challenges like these are not efficiently resolved, severe mental illness may develop, affecting productivity and safety in the workplace.

The East Campus of Shanghai Sixth People's Hospital, as the only third-level hospital in the Nanhui area, has assisted in diagnosing, investigating, and isolating the novel coronavirus infections in the Pudong area since the beginning of the epidemic. Under these circumstances, the medical staff faced tremendous physical and psychological pressure, giving rise to mental health issues. During the early stage of the epidemic, the Trade Union department of the hospital considered the psychological challenges employees were facing and launched the Employee Assistance Program (EAP), which includes different forms of psychological intervention (7). The EAP is a systematic and long-term assistance and welfare program for employees. Through the program, mental health professionals diagnose individual employees, advise the hospital organization, and provide professional guidance, training, and consultation.

In this study, we used the Symptom Checklist 90 (SCL-90) to evaluate the psychological well-being of hospital staff members (8). The SCL-90 is one of the most well-known mental health test scales in the world (9) and measures nine aspects of psychological well-being. Next, we analyzed the psychological changes of employees during different periods of the epidemic. We also carried out a unified analysis of staff members with higher SCL-90 scores to assess the epidemic's effects on individuals with mental illnesses.

The Hospital Trade Union considered the psychological state of medical staff members based on their SCL-90 scores and, according to severity, adopted different forms of psychological interventions. For staff members with SCL-90 scores indicating severe mental illness, the EAP intervened individually through effective professional psychological counseling measures, including small class videos and WeChat public classes. We then analyzed changes in the employees' psychological states before and after the EAP psychological intervention and further explored the psychological impacts of the epidemic on healthcare professionals.

## Research Methods

### Ethics

This study was approved by the Ethics Committee of the Eastern Campus of the Sixth People's Hospital of Shanghai, with ethics approval No. 201807-11. All participants provided informed consent before taking the survey.

### Subjects

The inclusion criteria were: the staff at our hospital who consented to questionnaires and psychological intervention. A total of 1,198 medical workers were included in this study from the hospital. All questionnaires were reported online by voluntary participation.

### Study Design

**Figure 1:**
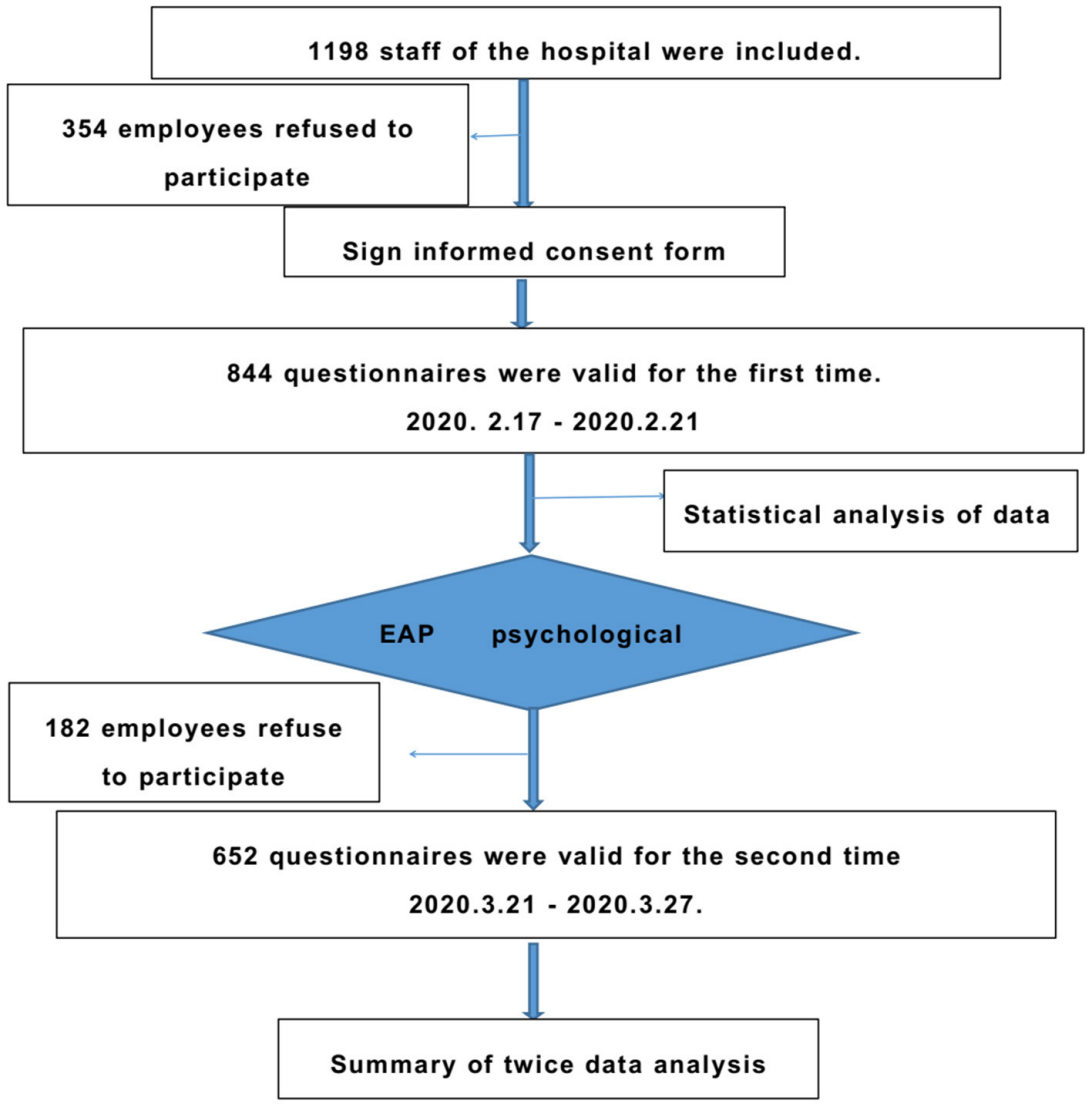
Research design flowchart.

This study used interventional clinical observation and psychological evaluation before and after the intervention. The study design flow chart is shown in Figure 1.

### Psychological Evaluation

Psychological well-being was evaluated by the SCL-90, which includes nine factors (8): somatization, interpersonal sensitivity, obsessive-compulsive symptoms, anxiety, depression, hostility, paranoia, terror, and psychosis. The test quantifies the psychological status of individuals in the last week through a five-point Likert scale (8), with higher scores indicating more severe mental health symptoms: 1 = none, 2 = very light, 3 = moderate, 4 = heavy, and 5 = serious. The number of positive items in the SCL-90 is >43, and the score of any one of these items is >2 points. A score of >160 points is considered a positive score, while a total score of >200 points indicates obvious psychological problems. A total score of >250 points indicates that psychological help is needed immediately.

### The SCL-90 Questionnaire

The SCL-90 questionnaire was prepared for this study through the https://www.wjx.cn/ website and its reliability and validity are analyzed. Based on a literature search (10) and expert consultation, a general questionnaire was created based on specific circumstances due to the epidemic that may affect the psychological well-being of hospital workers. In addition to specific questions related to the psychological status, the survey also asked about a healthcare worker's age, sex, education, length of service, professional title, position, type of post, number of siblings, marital status, fertility, parents, and previous first-line work experience with an epidemic (such as SARS). According to the degree of close contact with COVID-19 patients, positions were divided into seven types: aid to Hubei, the Shanghai Public Health Center, the fever clinic, internal medicine, surgery, pediatrics, and the emergency department. Hospital staff members answered the survey questions by scanning the QR code for 5 days; the platform's link was closed on the sixth day to collect the data. The contents of the questionnaires in the subsequent follow-up were the same.

### EAP Psychological Intervention

We analyzed and summarized the data from employees whose SCL-90 score was >160, and especially those with scores >200. The total symptom index was ≥2, and the number of positive items was ≥43. Since employee participation was completely voluntary and privacy was ensured, the hospital union adopted the EAP psychological assistance program to provide group intervention or individual intervention as follows (1) For individuals with a total score of >250, the hospital adopted a one-on-one psychological counseling service to provide a personal intervention with strict privacy. (2) The hotline was accessible to provide psychological assistance for public welfare (hotline: 021-64376570, 021-34141539, available Monday–Friday, 9:00–22:00). (3) The hospital organized different resources: a free EAP psychological website, psychological training, counseling through a series of micro-courses (once or twice a week for 15 min each), and WeChat meetings (conducted in small groups).

### Statistical Analysis

The data were analyzed by SPSS 22.0, the counting data were expressed as a percentage, and the measurement data were expressed as $\bar{\text{x}}\pm s$ ± s. Chi-square test, *t*-test, U test, Welch's test, and the Brown–Forsythe test were used for comparison between groups. A multinomial logistic regression model was used for multivariate analysis. *P* < 0.05 indicated that the difference was statistically significant.

## Results

### Baseline Information

**Figure 2:**
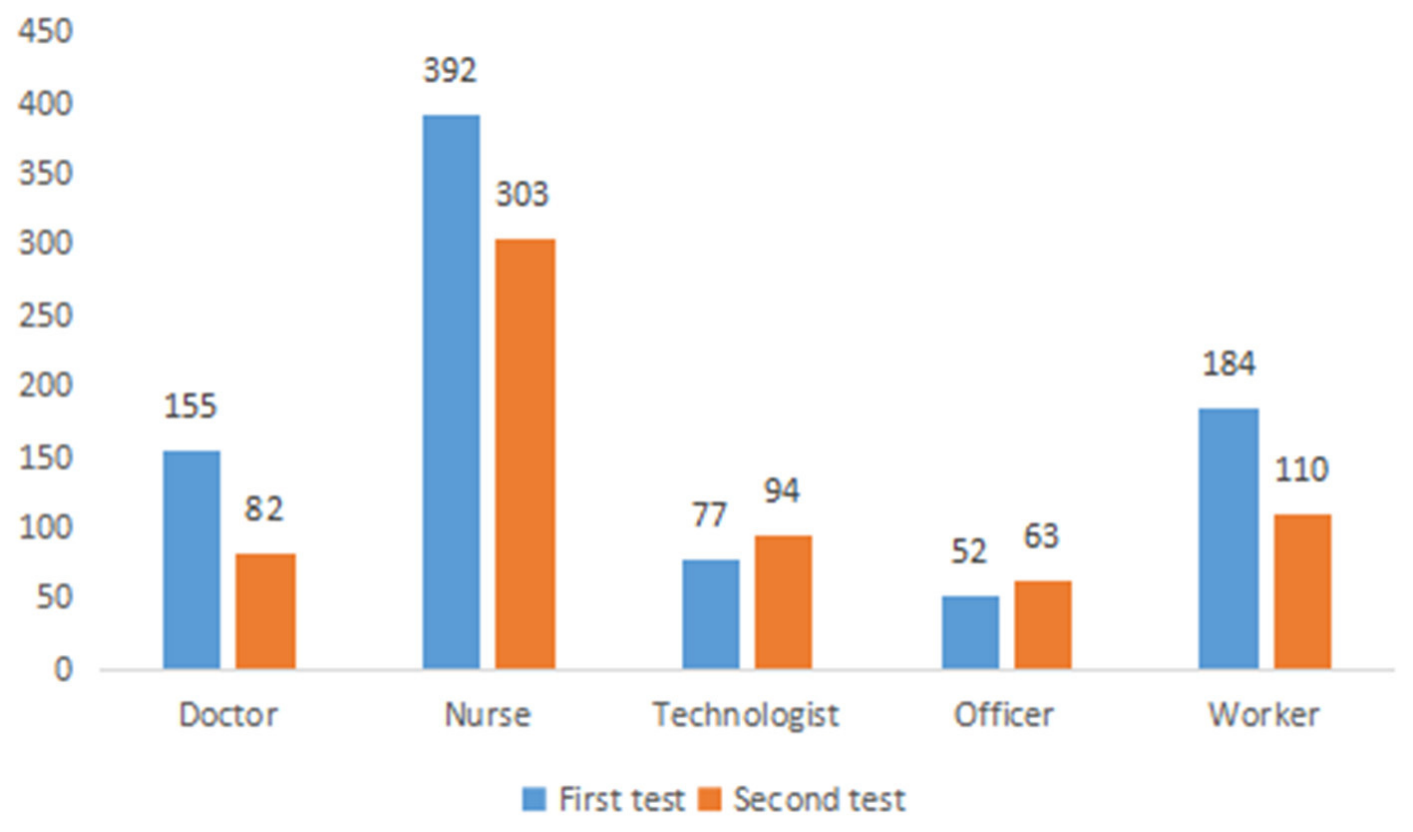
Comparison of the number of people in different majors participating in the two assessment.

The first questionnaire was distributed from February 17–21, 2020. The second questionnaire was distributed from March 21–27, 2020. Regarding the initial assessment, a total of 844(70.5%) valid questionnaires were collected, which included 155 doctors (18.02%), 392 nurses (45.58%), 77 personnel with medical skills (8.95%), 52 administrative staff members (6.05%), and 184 other hospital workers (21.4%). Regarding the second evaluation, 652(54.4%) valid questionnaires were collected, including 82 doctors (12.58%), 303 nurses (46.47%), 94 personnel with medical skills (14.42%), 63 administrative staff members (9.66%), and 110 other hospital workers (16.87%). Thus, the participation of doctors and other hospital workers decreased, while that of the technical staff increased. See Figure 2. Reliability and Validity Table 1 shows the SCL-90 questionnaire reliability and validity.

**Table 1:** Validity and reliability of the SCL-90 questionnaire.

**Item**	**Somatization**	**Obsessive** **compulsive**	**Interpersonal sensitivity**	**Depression**	**Anxiety**	**Hostile**	**Terror**	**Paranoia**	**Psychosis**
**Whole sample (** ***n*** **=** **844)**									
Cronbach alpha coefficient	0.83	0.81	0.89	0.81	0.80	0.73	0.79	0.78	0.80
Test-retest correlateion	0.86	0.83	0.77	0.93	0.84	0.78	0.80	0.89	0.77
Correlation with whole score	0.82	0.88	0.80	0.85	0.85	0.79	0.82	0.85	0.82

### Univariate Analysis of Related Factors of Employees With Abnormal Total Scores From the SCL-90 Assessment of Survey 1

The distribution of the total scores of all hospital workers participating in the survey 1 was > 160 points, as well as the specific basic situation analysis. The basic situation analysis is shown in Table 2. In terms of gender, the abnormal psychological symptoms rate of female employees was significantly higher than that of male employees (*P* = 0.005). Also, we found a significant difference when considering age: the highest rate of psychological abnormality was found in employees between 30 and 39 years old (*P* < 0.001). In addition, SCL-90 scores differed significantly based on occupation in the hospital (*P* < 0.001), with the abnormal psychological symptoms rate of nurses being the highest. Considering the first evaluation, the positive rate (i.e., an SCL-90 score of > 160) of medical staff working in Hubei and supporting public health centers was 100%, which was significantly alleviated after EAP intervention. The abnormal psychological symptoms rate of workers in the fever clinic was still high, and a significant correlation was established between posts and scores (*P* < 0.001). There were also significant differences between the test in terms of academic degrees (*P* < 0.001) and professional titles (*P* = 0.008); individuals with intermediate titles scored the highest. In terms of marriage, the abnormal psychological symptoms rate for unmarried employees was the highest after the first evaluation (*P* < 0.001).

**Table 2:** Basic situation of the subjects of the two surveys [total number of people (positive number > 160 points)].

	**Group**	**Frist research** **(***n*** = 844)**	**χ^2^**	***P*** **-value**	**Second research** **(***n*** = 652)(n,%)**	**χ^2^**	***P*** **-values**
Sex	Male	202 (23,11%)	7.889	0.005	126 (11,8%)	8.911	0.005
	Female	642 (129,20%)			526 (102,19%)		
Age (years)	29	248 (36,14%)	18.496	0.001	143 (25,17%)	2.153	0.074
	30–39	376 (91,24%)			353 (70,19%)		
	40–49	133 (16,12%)			102 (12,11%)		
	≥50	87 (9,10%)			54 (6,11%)		
Length of service	10	463 (86,18%)	2.022	0.364	366 (62,16%)	1.206	0.193
	10–19	220 (43,19%)			178 (35,19%)		
	20–29	101 (15,14%)			66 (7,10%)		
	≥30	60 (8,13%)			42 (9,21%)		
Professional position	Doctor	154 (24,15%)	38.794	0.001	82 (13,15%)	29.891	0.001
	Nurse	387 (99,25%)			303 (72,23%)		
	Technologist	77(10,12%)			94 (6,6%)		
	Office	51 (11,21%)			63 (16,25%)		
	Worker	175 (8,4%)			110 (6,5%)		
Post	Aid Hubei	4 (4,100%)		0.001	4 (2,50%)	24.516	0.004
	Aid PHC	4 (4,100%)			4 (1,25%)		
	Fever Clinic	24 (20,83%)			24 (18,75%)		
	Other post	812 (124,15%)			620 (191,30%)		
Degree	Doctor	28 (4,14%)	28.580	0.001	23 (3,13%)	39.845	0.001
	Master	137 (24,17%)			98 (20,20%)		
	Bachelor	348 (80,22%)			300 (61,20%)		
	College	192 (40,20%)			25 (5,20%)		
	Senior	139 (4,2%)			93 (4,4%)		
Professional title	Senior	65 (11,16%)	13.278	0.008	38 (5,12%)	42.689	0.006
	Intermediate	117 (25,21%)			87 (22,25%)		
	Junior	431 (92,21%)			365 (69,18%)		
	No	231 (24,9.8%)			162 (17,10%)		
Marital status	No	171 (121,70%)	46.577	0.001	508 (89,17%)	1.203	0.401
	Yes	649 (27,3%)			126 (19,15%)		
	Divorce	24 (4,16%)			18 (5,27%)		
Only one kid	Yes	373 (77,20%)	3.140	0.076	294 (61,20%)	5.265	0.037
	No	471 (75,15%)			358 (52,14%)		
Fertility situation	No	240 (36,15%)	73.929	0.499	185 (26,14%)	52.415	0.230
	One kid	476 (86,18%)			372 (66,17%)		
	Two or more	128 (30,23%)			95 (21,22%)		
Parents' situation	Parents	701 (128,18%)	0.173	0.675	544 (100,18%)	0.215	0.112
	Single family	143 (24,16%)			108 (13,12%)		
Joining SARS	Yes	90 (17,18%)	0.053	0.843	100 (19,19%)	0.078	0.632
	No	754 (136,18%)			552 (94,17%)		

### Multivariate Logistic Regression Analysis of Influencing Factors of Psychological Status

Taking mental health status as the dependent variable (a total score of more than 160 points is a positive mental health symptom), age, gender, length of service, occupation, education, professional title, marital status, whether an only child, fertility status, parent status, and whether there is experience working first-line in a major epidemic as independent variables, multivariate logistic regression analysis was performed, and the variable assignments are shown in Table 3. Multivariate logistic regression analysis found that the psychological state of hospital staff was correlated with gender, education, and fertility (*P* < 0.05). The results showed that compared with women, men's mental health status was better, with an OR value of 0.598 (0.372–0.962). Groups with high school education levels, junior high school, or below were at higher risk of psychological problems, with OR values of 23.655 (2.815–198.784) and 9.09 (2.601–31.801), respectively. Administrative occupations and having two or more children were protective factors for mental health, and the OR values were 0.400 (0.175–0.912) and 0.327 (0.152–0.703), respectively (Table 4).

**Table 3:** Logistic regression analysis variable assignment.

**Variable**	**Assignment**
Mental health status	Positive symptoms=1; Normal=0
Age	<30 years old = 1; 30–39 years old = 2; 40-49 years old = 3; ≥50 years old = 4
Gender	Female = 1; Male = 2
Length of service	<10 years = 1;10–19 years = 2; 20-29 years = 3; ≥30 years = 4
Occupation	Doctor = 1; Nurse = 2; Medical technician = 3; Office staff = 4; Worker = 5
Educational background	Master's degree and above = 1; Bachelor's degree = 2; Associate degree = 3; High school = 4; Junior high school and below = 5
Professional title	Advanced = 1; Intermediate = 2; Primary = 3; None = 4
Marital status	Married = 1; Unmarried = 2; Divorced or other = 3
Whether the only child	Yes = 1; No = 2
Fertility status	No childbearing =1; One child = 2; Two children and more = 3
Parent status	Both parents = 1; Single parent or others = 2
Whether there is a major epidemic first-line work experience	Yes = 1; No = 2

**Table 4:** Logistic multivariate analysis of factors affecting the mental health of hospital staff.

**Factor**	**β**	**S.E**	**Wald** ***χ*** **^2^**	***P* value**	**OR**	**(95%CI)**	**Tolerance**	**VIF**
**Gender**	−0.514	0.243	4.489	0.034	0.598	0.372–0.962	0.308	3.247
**Age (year)**								
<30 (reference)								
30~	−0.344	0.255	1.819	0.177	0.709	0.430–1.169	0.421	2.375
40~	0.752	0.575	1.708	0.191	2.121	0.867–6.554	0.236	4.237
≥50	0.108	0.779	0.019	0.889	1.114	0.242–5.128	0.125	8
**Length of service (year)**								
<10 (reference)								
10~	0.341	0.236	2.096	0.148	1.407	0.886–2.232	0.352	2.84
20~	−1.010	0.567	3.175	0.075	0.364	0.120–1.106	0.251	3.984
≥30	−0.091	0.394	0.053	0.818	0.913	0.422–1.975	0.260	3.846
**Occupation**								
Doctor (reference)								
Nurse	−0.708	0.420	2.841	0.092	0.493	0.216–1.122	0.365	2.74
Medical technician	−0.248	0.423	0.344	0.558	0.780	0.341–1.787	0.524	1.908
Office staff	−0.916	0.420	4.749	0.029	0.400	0.175–0.912	0.700	1.429
Worker	−0.021	0.599	0.001	0.973	0.980	0.303–3.617	0.254	3.937
**Educational background**								
Master's degree and above (reference)								
Bachelor's degree	−0.013	0.262	0.003	0.959	0.987	0.590–1.649	0.652	1.534
Associate degree	0.009	0.306	0.001	0.978	1.009	0.554–1.836	0.520	1.923
High school	3.164	1.086	8.485	0.004	23.655	2.815–198.784	0.321	3.115
Junior high school and below	2.208	0.639	11.948	0.001	9.095	2.601–31.801	0.562	1.78
**Professional title**								
Advanced (reference)								
Intermediate	−0.346	0.474	0.534	0.465	0.708	0.280–1.790	0.454	2,203
Primary	−0.358	0.496	0.522	0.470	0.699	0.264–1.847	0.285	3.509
None	−0.192	0.544	0.124	0.725	0.826	0.284–2.399	0.641	1.56
**Marital status**								
Married (reference)								
Unmarried	−0.095	0.370	0.065	0.798	0.910	0.440–1.879	0.785	1.274
Divorced or other	−0.288	0.506	0.324	0.569	0.750	0.278–2.021	0.491	2.037
Whether the only child	0.190	0.179	1.126	0.289	1.209	0.851–1.719	0.454	2,202
**Fertility status**								
No childbearing (reference)								
One child	−0.548	0.333	2.710	0.100	0.578	0.301–1.110	0.235	4.255
Two children and more	−1.118	0.390	8.206	0.004	0.327	0.152–0.703	0.641	1.56
Parent status	−0.275	0.252	1.192	0.275	0.760	0.464–1.244	0.295	3.39
Whether there is a major epidemic first-line work experience	−0.045	0.272	0.027	0.869	0.956	0.561–1.629	0.215	4.651

### EAP Psychological Intervention Service

The scores of the two evaluations are shown in Table 5. Employees with a score of > 250 in their initial psychological assessment consisted of three doctors, eight nurses, one office worker, and one medical technologist. Voluntary one-on-one assistance was offered to these 13 people. The counseling meeting was held twice for a small group of < 15 individuals, while hospital-wide counseling was held only once. Psychological counseling via telephone was open indefinitely. After the first evaluation, a total of 11 individuals participated in the individual evaluation, 22 participated in small-scale psychological counseling, 30 participated in voluntary telephone counseling, and 680 participated in WeChat and network counseling. After the second evaluation, 10 personnel, including 2 doctors and 8 nurses, presented a score of > 250. The two doctors were among three that were evaluated for the first time. Eight nurses were new (first assessment score was 201–250). The scores of employees participating in different intervention methods were compared and analyzed, as shown in Table 6. After one-to-one counseling, total score of SCL-90, Obsessive compulsive, Depression, Anxiety, Terror were significantly decreased (*P* < 0.05). After small group consultation, total score of SCL-90, Obsessive compulsive, Depression, and Anxiety were significantly decreased (*P* < 0.05). After telephone consultation, the total score of SCL-90, Depression and Anxiety were significantly decreased (*P* < 0.05). Obsessive compulsive, Depression, and Anxiety decreased significantly after WeChat or online counseling (*P* < 0.05).

**Table 5:** Total SCL-90 score distribution of healthcare professionals.

		**Doctor**	**Nurse**	**Office**	**Technologist**	**Worker**	**Total**
>250	First	3	8	1	1	0	13
	Second	2	8	0	0	0	10
201–250	First	7	15	3	2	2	29
	Second	3	14	4	1	2	24
160–200	First	17	53	7	7	6	90
	Second	9	51	11	5	4	80
<160	First	128	291	41	67	176	603
	Second	68	230	47	88	104	537

**Table 6:** Comparison of M ± SD scores of employees in different positions after the intervention.

	**Total scores**	**Somatization**	**Obsessive compulsive**	**Interpersonal sensitivity**	**Depression**	**Anxiety**	**Hostile**	**Terror**	**Paranoia**	**Psychosis**
A	271.6 ± 39.44	2.99 ± 0.61	3.08 ± 0.78	3.16 ± 0.32	2.83 ± 0.58	3.45 ± 0.25	2.77 ± 0.44	2.66 ± 0.58	2.69 ± 0.49	2.84 ± 0.55
*n* = 11	259.1 ± 30.20	2.80 ± 0.29	2.53 ± 0.43	3.38 ± 0.42	2.41 ± 0.44	3.08 ± 0.40	2.52 ± 0.26	2.19 ± 0.35	2.54 ± 0.39	2.75 ± 0.49
*t*	32.21	1.25	11.95	0.25	21.69	15.69	−1.252	23.65	0.632	1.136
*p*	0.002	0.356	0.001	0.895	0.021	0.003	0.596	0.016	0.524	0.256
B	221.6 ± 30.14	2.80 ± 0.21	2.98 ± 0.38	3.06 ± 0.32	2.88 ± 0.58	2.85 ± 0.25	2.57 ± 0.44	2.56 ± 0.58	2.49 ± 0.49	2.64 ± 0.35
*n* = 22	201.1 ± 19.20	2.41 ± 0.49	2.03 ± 0.23	2.98 ± 0.42	2.51 ± 0.44	2.58 ± 0.40	2.42 ± 0.26	2.43 ± 0.35	2.40 ± 0.39	2.36 ± 0.49
*t*	37.52	−2.256	20.23	2.56	15.69	25.36	0.256	2.67	0.215	0.985
*p*	0.011	0.085	0.012	0.083	0.010	0.004	0.152	0.065	0.852	0.378
C	190.68 ± 38.12	2.22 ± 0.62	2.49 ± 0.41	2.43 ± 0.36	2.51 ± 0.32	2.44 ± 0.35	2.06 ± 0.38	2.24 ± 0.42	2.43 ± 0.21	2.41 ± 0.17
*n* = 30	172.43 ± 34.82	2.44 ± 0.38	2.38 ± 0.38	2.39 ± 0.24	2.29 ± 0.14	2.40 ± 0.30	2.13 ± 0.28	2.15 ± 0.36	2.33 ± 0.20	2.33 ± 0.25
*t*	8.256	−1.254	0.986	1.591	9.689	1.200	−1.268	0.547	0.215	0.259
*p*	0.035	0.156	0.085	0.055	0.027	0.059	0.085	0.259	0.781	0.986
D	132.47 ± 15.92	1.72 ± 0.28	1.49 ± 0.51	1.41 ± 0.47	1.58 ± 0.55	1.36 ± 0.41	1.37 ± 0.55	1.29 ± 0.35	1.41 ± 0.24	1.30 ± 0.26
*n* = 680	125.15 ± 19.05	1.31 ± 0.49	1.23 ± 0.31	1.25 ± 0.27	1.24 ± 0.23	1.21 ± 0.21	1.24 ± 0.22	1.16 ± 0.20	1.27 ± 0.29	1.27 ± 0.20
*t*	1.621	4.569	1.201	3.658	8.965	3.548	0.265	0.159	0.986	0.800
*p*	0.055	0.038	0.051	0.040	0.012	0.035	0.098	0.146	0.587	0.069

*A, one-on-one psychological consultation; B, small-scale group consultation; C, telephone consultation; D, WeChat or online consultation*.

### Comparative Analysis of the SCL-90 Scores Between Different Professional Occupations

For data that were not in accordance with the homogeneity of variance, a comparison of average scores of the SCL-90 factors in different professional positions (Table 7) was conducted through a Welch's test. The results showed that the total scores of workers in various positions differed significantly after two evaluations, and we observed a downward trend with statistical significance among the groups (*P* < 0.01). Moreover, differences were noted in somatization, obsessive-compulsive symptoms, interpersonal sensitivity, depression, anxiety, hostility, phobia, paranoia, and psychosis among the different categories of personnel (*P* < 0.01). After EAP training, we re-analyzed the paired scores of employees of different occupations who participated in the two evaluations, as shown in Table 8. This paired analysis showed that the depression and anxiety of the doctor group were significantly reduced. In the nursing group, the total score, somatization, interpersonal sensitivity, depression, and anxiety were significantly reduced. In the medical technician group, depression, anxiety, and paranoia were significantly reduced. Among office staff, no significant differences were found. Among workers, the total score, depression, and anxiety were significantly reduced.

**Table 7:** Total scores and scores based on each factor of the two SCL-90 evaluations.

	**Doctor**	**Nurse**	**Technologist**	**Officer**	**Worker**	***Z***	***P*-value**
Total scores	126.9 ± 29.18	138.68 ± 37.44	123.47 ± 29.92	134.75 ± 30.12	108.07 ± 16.93	42.58	<0.001
	125.1 ± 31.20	132.43 ± 34.82	112.15 ± 18.05	132.70 ± 31.71	105.84 ± 15.60	56.31	<0.001
Somatization	1.38 ± 0.31	1.53 ± 0.42	1.32 ± 0.28	1.54 ± 0.43	1.17 ± 0.19	94.126	< 0.001
	1.30 ± 0.29	1.44 ± 0.38	1.23 ± 0.19	1.49 ± 0.39	1.15 ± 0.17	59.179	< 0.001
Obsessive compulsive	1.57 ± 0.41	1.77 ± 0.50	1.56 ± 0.41	1.54 ± 0.46	1.37 ± 0.33	70.710	< 0.001
	1.53 ± 0.43	1.68 ± 0.48	1.43 ± 0.31	1.46 ± 0.44	1.31 ± 0.27	56.545	< 0.001
Interpersonal sensitivity	1.45 ± 0.40	1.53 ± 0.46	1.38 ± 0.37	1.58 ± 0.50	1.22 ± 0.24	48.376	< 0.001
	1.38 ± 0.42	1.49 ± 0.44	1.25 ± 0.27	1.54 ± 0.43	1.20 ± 0.24	50.077	< 0.001
Depression	1.46 ± 0.40	1.57 ± 0.48	1.38 ± 0.35	1.58 ± 0.47	1.21 ± 0.21	66.371	< 0.001
	1.41 ± 0.44	1.49 ± 0.44	1.24 ± 0.23	1.57 ± 0.40	1.17 ± 0.20	61.452	< 0.001
Anxiety	1.39 ± 0.35	1.53 ± 0.45	1.35 ± 0.30	1.34 ± 0.40	1.16 ± 0.17	89.469	< 0.001
	1.38 ± 0.40	1.44 ± 0.40	1.21 ± 0.21	1.32 ± 0.36	1.14 ± 0.18	66.874	< 0.001
Hostile	1.38 ± 0.34	1.55 ± 0.48	1.38 ± 0.35	1.48 ± 0.36	1.17 ± 0.19	69.966	< 0.001
	1.22 ± 0.26	1.53 ± 0.48	1.24 ± 0.22	1.43 ± 0.42	1.12 ± 0.17	82.026	< 0.001
Terror	1.33 ± 0.38	1.43 ± 0.42	1.23 ± 0.25	1.39 ± 0.38	1.13 ± 0.18	71.832	< 0.001
	1.29 ± 0.35	1.35 ± 0.36	1.16 ± 0.20	1.37 ± 0.33	1.12 ± 0.18	53.025	< 0.001
Paranoia	1.30 ± 0.31	1.40 ± 0.41	1.31 ± 0.34	1.46 ± 0.42	1.14 ± 0.19	50.212	< 0.001
	1.24 ± 0.29	1.38 ± 0.40	1.17 ± 0.19	1.43 ± 0.35	1.12 ± 0.18	50.185	< 0.001
Psychosis	1.28 ± 0.30	1.39 ± 0.37	1.28 ± 0.26	1.47 ± 0.38	1.14 ± 0.17	49.524	< 0.001
	1.25 ± 0.29	1.33 ± 0.35	1.17 ± 0.20	1.41 ± 0.35	1.14 ± 0.18	45.706	< 0.001

**Table 8:** Total scores and scores based on each factor of the two SCL-90 evaluations(M ± SD).

	**Total scores**	**Somatization**	**Obsessive compulsive**	**Interpersonal sensitivity**	**Depression**	**Anxiety**	**Hostile**	**Terror**	**Paranoia**	**Psychosis**
D	127.6 ± 29.44	1.40 ± 0.41	1.64 ± 0.48	1.46 ± 0.52	1.48 ± 0.48	1.45 ± 0.65	1.37 ± 0.44	1.28 ± 0.58	1.25 ± 0.41	1.26 ± 0.45
*n* = 82	125.1 ± 31.20	1.34 ± 0.29	1.63 ± 0.43	1.38 ± 0.42	1.21 ± 0.44	1.31 ± 0.40	1.22 ± 0.26	1.29 ± 0.35	1.24 ± 0.29	1.25 ± 0.29
*t*	1.010	0.258	0.985	1.032	5.985	6.254	0.895	−1.251	0.659	1.891
*p*	0.596	0.635	0.321	0.658	0.012	0.026	0.115	0.895	0.956	0.625
N	140.68 ± 38.12	1.57 ± 0.62	1.69 ± 0.61	1.53 ± 0.56	1.61 ± 0.42	1.54 ± 0.65	1.56 ± 0.78	1.44 ± 0.52	1.43 ± 0.51	1.41 ± 0.77
*n* = 303	131.43 ± 34.82	1.24 ± 0.38	1.68 ± 0.48	1.39 ± 0.44	1.49 ± 0.44	1.44 ± 0.40	1.53 ± 0.48	1.45 ± 0.36	1.38 ± 0.40	1.33 ± 0.35
*t*	8.592	3.698	0.621	2.215	5.685	3.658	0.254	−1.185	0.584	0.208
*p*	0.025	0.042	0.085	0.035	0.037	0.014	0.897	0.968	0.857	0.587
T	123.47 ± 29.92	1.32 ± 0.28	1.49 ± 0.51	1.41 ± 0.47	1.41 ± 0.55	1.36 ± 0.41	1.37 ± 0.55	1.29 ± 0.55	1.41 ± 0.24	1.30 ± 0.46
*n* = 77	121.15 ± 19.05	1.31 ± 0.49	1.43 ± 0.31	1.35 ± 0.27	1.24 ± 0.23	1.21 ± 0.51	1.34 ± 0.22	1.26 ± 0.20	1.17 ± 0.19	1.27 ± 0.20
*t*	0.985	0.154	1.021	0.754	3.587	1.852	0.852	0.569	4.652	0.875
*p*	0.598	0.698	0.962	0.658	0.041	0.043	0.365	0.125	0.035	0.195
O	134.75 ± 30.12	1.54 ± 0.43	1.54 ± 0.46	1.58 ± 0.50	1.58 ± 0.47	1.34 ± 0.40	1.48 ± 0.36	1.39 ± 0.38	1.46 ± 0.42	1.47 ± 0.38
*n* = 51	130.70 ± 34.71	1.44 ± 0.62	1.51 ± 0.40	1.53 ± 0.31	1.56 ± 0.49	1.31 ± 0.26	1.44 ± 0.49	1.36 ± 0.39	1.44 ± 0.15	1.42 ± 0.65
*t*	1.251	0.957	0.121	1.230	0.845	0.966	1.012	0.854	0.857	0.555
*p*	0.986	0.081	0.658	0.945	0.178	0.845	0.256	0.965	0.563	0.981
W	110.07 ± 16.03	1.18 ± 0.29	1.39 ± 0.53	1.26 ± 0.44	1.24 ± 0.41	1.19 ± 0.37	1.20 ± 0.49	1.14 ± 0.28	1.19 ± 0.31	1.15 ± 0.27
*n* = 110	105.84 ± 15.60	1.15 ± 0.17	1.31 ± 0.27	1.20 ± 0.24	1.17 ± 0.20	1.14 ± 0.18	1.12 ± 0.17	1.12 ± 0.18	1.17 ± 0.18	1.14 ± 0.18
*t*	2.532	0.952	0.232	0.521	1.582	0.852	0.365	0.254	0.584	0.977
*p*	0.042	0.112	0.895	0.691	0.036	0.054	0.874	0.986	0.258	0.685

*D, doctorss; N, nurses; T, technicians; O, office staff; W, workers*.

## Discussion

This study evaluated hospital staff members undertaking the crucial medical tasks surrounding the novel coronavirus. The cohort study and analysis revealed psychological changes of hospital employees in different epidemic periods. The SCL-90 scores of some posts were higher than that of others, as demonstrated through various forms of EAP psychological assistance. Our findings reveal that professional psychological counseling could potentially alleviate psychological illnesses, provide a theoretical basis for promoting EAP psychological services in China, and demonstrate how to reduce pressure on hospital staff members during an epidemic. These implications are important, considering that these key factors could ultimately help overcome the epidemic.

At the beginning of the epidemic, healthcare workers on the front lines knew little about the novel coronavirus; however, their experience increased gradually over time (11). Because the risk of occupational exposure to the novel coronavirus is high for front-line medical workers, they also face immense psychological pressure. These healthcare professionals have devoted themselves to heavy treatment work, which required them to be in frequent contact with patients' blood, bodily fluids, and sharp medical instruments, while their psychological preparation and knowledge about the virus were limited (12). During working hours, medical staff members wear protective equipment that restricts their ability to breathe normally, and they also experience other challenges such as physical and mental fatigue. When they are not working, their time to rest is in hotels, which is isolating. Their everyday lives are limited, they cannot meet with relatives and friends, and their social support is insufficient (13). Given all of these factors, medical workers have had to endure a significant amount of physical and mental stress during the epidemic.

In this study, we found that the abnormal psychological symptoms rate (i.e., an SCL-90 score of >160) of the first evaluation reached 18.26%, which was similar to the psychological abnormality of medical workers in Wuhan during the current novel coronavirus epidemic (14). Promptly identifying and resolving the abnormal psychological state of medical staff members is essential to combat the epidemic. Trade unions in the hospital actively provide free EAP psychological support services, thereby serving as a major source of support during the epidemic.

EAP services also play a major role in many medical units worldwide (15). In this epidemic, our EAP service provided a meaningful platform to relieve the psychological pressure for the majority of medical staff members. During the early stages of the novel coronavirus outbreak, the psychological burden of self-assessment and the enthusiasm to participate in the SCL-90 assessment were both high. Over time, China's control of the novel coronavirus is improving, and the incidence rate is declining gradually. The hospital staff, especially doctors, have also gained confidence in controlling the epidemic, and participation in the SCL-90 assessments has declined. In analyzing hospital employees with a total SCL-90 score of >160, significant differences were detected when considering factors such as age, gender, educational background, professional title, position, post category, and marital status. We found that employees whose SCL-90 scores indicated abnormal psychological symptoms tended to be women, workers between 30 and 39 years old, nurses, undergraduates, employees with intermediate professional titles, and unmarried individuals; as such, our EAP psychological assistance targets were also concentrated on these employees. The mental health status of men is better than that of women, which might be related to the different social roles and life pressures borne by different genders. Typically, it is believed that women's psychological ability to deal with crises is on the low side (16). Regarding levels of education, researchers have found that high educational backgrounds are correlated with improved mental health status due to the maturing of the psychological defense mechanism, resulting in greater self-control and social adaptability (17). In this study, the abnormal psychological symptoms rate of nurses' scores was considerably high. This finding could be related to nurses constituting a major subset of the healthcare workers on the front lines of the epidemic. China's fertility situation is different from that of other countries because of the two-child policy, which was instituted in 2015 (18). The present study found that a hospital worker's number of children was not correlated with his or her abnormal mental state.

China has focused on the psychological counseling of medical staff and psychological disorders during epidemics such as SARS, and earlier psychological interventions have led to better results (19). During the novel coronavirus epidemic, psychological care was provided early in many parts of China, especially Hubei; many medical teams in Hubei are equipped with psychiatrists (20). EAP organizations have played a critical role in alleviating employees' psychological problems internationally (21). For example, our hospital introduced an EAP psychological counseling team in 2015. For group intervention, we used the hospital's official WeChat account for collective training and focusing on online activities. Specifically, the emotional intelligence group intervention improved the emotional intelligence levels of the medical staff and their quality of life. This form of intervention could also be used for psychological quality training and stress management of the medical staff.

Because our counseling was conducted voluntarily, only 11 individuals were willing to receive one-on-one counseling. The majority of the medical staff chose to participate in group training, online resources, or telephone counseling.

Approximately 300 million people worldwide have mild to moderate mental illnesses, but not all individuals can recognize their illnesses (22). Chinese studies have found that the biggest enemy of the popularization of psychological services is a bias in the public's perception of receiving psychological care; shame is a barrier between psychological counseling workers and the general public (23). In this regard, the results of our survey are consistent with other Chinese studies. Furthermore, even if psychological illnesses are identified, not all individuals choose to actively seek help. In this epidemic, a large-scale psychological survey of medical staff in Hunan also found that medical staff members are unwilling to face their psychological illnesses or challenges and feel that receiving psychological counseling is embarrassing (24). Thus, medical institutions should strengthen the psychological skills training of medical staff members so that hospitals can alleviate patients' anxiety, panic, and other psychological problems and improve the psychological well-being of their healthcare professionals. Increasing social support and setting up psychological intervention groups for medical staff members with anxiety and stress disorders is also a helpful and actionable step that hospitals can take, with the earliest interventions being ideal (25).

In this survey, we found that after a positive psychological intervention, the number of individuals with an SCL-90 total score of >160 decreased significantly. We also found that before EAP, the scores of obsessive-compulsive symptoms of hospital workers in various occupations were high, and among the nurses, the overall score was highest. Furthermore, the anxiety of doctors in Hubei and the fever clinics was severe, indicating the necessity of actively providing EAP psychological assistance to these high-risk groups. After the EAP provided psychological assistance to hospital workers, each factor score was lower than that of the initial assessment, indicating relief in all aspects of psychological illness. This finding also shows that actively providing and encouraging employees to participate in EAP psychological counseling can significantly alleviate abnormal psychological symptoms.

A limitation of this study is that it is observational; hence, we could not analyze the true causes of severe psychological symptoms through a survey. Our research has the inevitable defects of an online survey, such as the limitation of questionnaire design, the difficulty in guaranteeing the sample size, the accuracy of demographic information, and the cheating behavior of participants. Moreover, some important data was not collected, such as work performance-related outcomes and how employees perceive their employers' resources. Finally, in follow-up observations, only a small proportion of healthcare professionals were willing to participate in one-on-one psychological help and the intervention time was short. Considering the second evaluation, some medical personnel thought that they had no problems and refused to participate, resulting in a reduction in the data for analysis. Therefore, the current results can only reflect some of the psychological changes that occurred during this period. Increasing the publicity of psychological counseling could increase the number of people willing to receive psychological assistance. Thus, the EAP could help a larger number of professionals. In addition, the limitation of this article is the use of electronic questionnaires. The paper version of the questionnaire is conducive to more detailed thinking of the questionnaire respondents, and at the same time it is more widely used. However, during the epidemic, in order to reduce the gathering of people, we adopted the form of electronic questionnaires. In order to avoid wasting resources, we repeatedly trained and informed participants before the survey.

This study supports the practice of hospitals providing psychological assistance to high-risk medical staff in the future via an optimized plan, especially in the event of a sudden and major epidemic. These services have the potential to help healthcare professionals manage their mental health while fighting against the novel coronavirus epidemic.

## Data Availability Statement

The original contributions presented in the study are included in the article/supplementary material, further inquiries can be directed to the corresponding author/s.

## Ethics Statement

This study was approved by the ethics committee of the Eastern Campus of the Sixth People's Hospital of Shanghai. All participants provided informed consent before taking the survey.

## Author Contributions

JX, XL, and YX: the overall design and data analysis of the project and the revision of the article. YC and JZ: data collection. XF and YC: data analysis. XF, YC, and JZ: article writing. All authors contributed to the article and approved the submitted version.

## Conflict of Interest

The authors declare that the research was conducted in the absence of any commercial or financial relationships that could be construed as a potential conflict of interest.

## Publisher's Note

All claims expressed in this article are solely those of the authors and do not necessarily represent those of their affiliated organizations, or those of the publisher, the editors and the reviewers. Any product that may be evaluated in this article, or claim that may be made by its manufacturer, is not guaranteed or endorsed by the publisher.
